# Modelling the prognostic effect of glucose and lipid profiles on stroke recurrence in Malaysia: an event-history analysis

**DOI:** 10.7717/peerj.8378

**Published:** 2020-02-14

**Authors:** Xin Wee Chen, Mohd Nazri Shafei, Zariah Abdul Aziz, Norsima Nazifah Sidek, Kamarul Imran Musa

**Affiliations:** 1 Public Health Medicine Department, Faculty of Medicine, Universiti Teknologi MARA, Sungai Buloh, Selangor, Malaysia; 2 Department of Community Medicine, School of Medical Sciences, Universiti Sains Malaysia, Kubang Kerian, Kelantan, Malaysia; 3 General Medicine Department, Hospital Sultanah Nur Zahirah, Terengganu, Malaysia; 4 Clinical Research Center, Hospital Sultanah Nur Zahirah, Terengganu, Malaysia

**Keywords:** Survival analysis, Glucose, Lipids, Stroke, Recurrence

## Abstract

**Background:**

Diabetes and dyslipidemia are significantly associated with stroke recurrence, yet the evidence for this relationship is conflicting. Consequently, the parameters in the glucose and lipid profiles may inform us regarding their relationship with stroke recurrence, thus enhancing the physicians’ clinical decision-making.

**Aim:**

This study sought to investigate whether glucose and lipid profiles could prognosticate stroke recurrence in Malaysia.

**Methods:**

We conducted a retrospective hospital-based study where we analyzed the first-ever stroke cases regarding about which the Malaysia National Stroke Registry was informed between 2009 and 2017, that fulfilled this study’s criteria, and that were followed for stroke recurrence. Using the Cox proportional hazard regression analysis, we estimated the adjusted hazard ratios (HRs), which reflected the prognostic effect of the primary variables (i.e., glucose and lipid profiles on the first-stroke admission) on stroke recurrence.

**Results:**

Among the 8,576 first-ever stroke patients, 394 (4.6%) experienced a subsequent first stroke recurrence event. The prognostic effect measured by univariable Cox regression showed that, when unadjusted, ten variables have prognostic value with regards to stroke recurrence. A multivariable regression analysis revealed that glucose was not a significant prognostic factor (adjusted HR 1.28; 95% CI [1.00–1.65]), while triglyceride level was the only parameter in the lipid profile found to have an independent prognostication concerning stroke recurrence (adjusted HR: 1.28 to 1.36).

**Conclusions:**

Triglyceride could independently prognosticate stroke recurrence, which suggests the role of physicians in intervening hypertriglyceridemia. In line with previous recommendations, we call for further investigations in first-ever stroke patients with impaired glucose and lipid profiles and suggest a need for interventions in these patients.

## Introduction

Stroke recurrence refers to “a new neurologic deficit, including ischemic (or transient ischemic attack) or hemorrhagic stroke [that is] associated with rehospitalization” (Mi et al., 2012). Dyslipidemia and diabetes are widely established risk factors for stroke development and stroke recurrence, but the evidence for this association is inconsistent.

In Malaysia, total cholesterol has shifted its ranking as one of the leading risk factors for the attributable burden of disease from 12th to the 7th position between 1990 and 2015, respectively (GBD 2015 Risk Factors Collaborators, 2016). Furthermore, the various metabolic and cardiovascular risks, such as high fasting plasma glucose and high body mass index, have led to increased deaths and reduced disability-adjusted longevity from 2005 to 2015 (GBD 2015 Risk Factors Collaborators, 2016; Ministry of Health Malaysia, 2017). Hence, the role of impaired glucose and lipid metabolism in prognosticating stroke recurrence is worth exploring to enhance secondary stroke prevention (Awada, 2011).

Previous studies have reported a significant relationship of impaired glucose metabolism (Chandratheva, Geraghty & Rothwell, 2011; Ghandehari et al., 2013; Jia et al., 2011; Mi et al., 2012) and lipid metabolism (Callaly et al., 2016; Feng et al., 2009; Kumral et al., 2014) with stroke recurrence. However, there have also been studies that found a non-significant relationship between hyperlipidemia (Ghandehari et al., 2013; Li et al., 2017; Liu et al., 2015) and diabetes (Li et al., 2017) in the predictive models. Atherogenic dyslipidemia, which is characterized by reduced high-density lipoprotein cholesterol (HDL), increased low-density lipoprotein cholesterol (LDL) and increased triglyceride level, has been identified as an important independent cardiovascular risk marker (Musunuru, 2010; Talayero & Sacks, 2011). Interestingly, limited number of available studies confirm the hypotheses derived from an exploratory approach based on the primary variables of local clinicians’ interest (Counsell & Dennis, 2001; Kutner et al., 2004). This study was the natural extension of the aforementioned published studies, which identified glucose and lipid metabolism as the prognostic factors in an exploratory analysis.

Several randomized clinical trials to date have reported consistent benefits exist with reducing total cholesterol and LDL for vascular risk (Ministry of Health Malaysia, 2017). It was also suggested that LDL be the primary serum lipid parameter considered for reducing stroke recurrence risk (Park, Lee & Ovbiagele, 2014). Besides that, the strong relationship between plasma triglyceride and cardiovascular risk was well-posed in another study; however, as this investigation recruited mostly white and male subjects (Schwartz et al., 2015), the relevancy of authors’ findings to other populations was indeterminate. Collectively, the association of the fasting lipid parameters and stroke recurrence may be attributable to their role in promoting the formation of fatty deposits in the arteries.

This study sought to investigate the outcome as well as the time to stroke recurrence using an event history analysis (i.e., regression of longitudinal event data), which is more informative when compared to a mere quantification of whether an event happened or not (George, Seals & Aban, 2014; Singer & Willett, 2003). We aimed to estimate the prognostic effects of glucose and lipid profiles on stroke recurrence among first-ever stroke patients in Malaysia using the nationwide registry.

## Material and Methods

This study was a multicenter, retrospective cohort study that employed the data collected from the Malaysia National Stroke Registry. This registry is a national, prospective ongoing cohort investigation conducted actively to collect non-mandatory notification of stroke admissions in Malaysian public hospitals (Nazifah et al., 2012; NNEUR, 2013). The study received approval from both the Human Ethics Committee of the Universiti Sains Malaysia (USM/JEPeM/1711-576) and the Medical Research and Ethics Committee of the Ministry of Health Malaysia (NMRR-17-2500-38534). Permission to access the registry database was obtained from the Registry Steering Committee. Confidentiality of the data was kept throughout the study. Only the researchers had access to the data.

### Case ascertainment

The target population were patients admitted to public hospitals. These patients’ data were eligible for analysis if they met the following recruitment criteria: (1) the data came from clinically diagnosed stroke subjects aged 18 and above, (2) the data were notified to the registry between July 2009 and December 2017, and (3) patients were documented as having a first episode of stroke when notified to the registry. Patients with a principal hospital discharge diagnosis of ischemic stroke, intracerebral hemorrhage and subarachnoid hemorrhage based on the International Statistical Classification of Diseases and Related Health Problems (10th Revision) (World Health Organization, 2015) were included, whereas those with a transient ischemic attack, unknown or unclassified stroke type were excluded from the study.

### Variables

Each subject was identified using their unique registration number (with the Malaysian National Stroke Registry), as well as their full name and date of birth to determine if they had a subsequent notification. If patients experienced several recurrence events, only data from the first recurrence episode available on the MNSR were taken. In assessing survival, the outcome variable was the time-to-stroke recurrence after the first-ever stroke throughout the study period. The survival time (in years) was measured from the date of the first-stroke diagnosis to the subsequent recurrence, during which both admissions were notified to the registry. The event was defined as a failure (stroke recurrence, coded as 1) or censored (coded as 0). Patients who were lost to follow-up, and who have not developed recurrence during the study period, were considered as censored observations in the time-to-event analysis (Singer & Willett, 2003). The primary variables referred to the glucose and lipid profiles. Abnormal glucose level was defined with the cut-off values of glucose in the emergency department (ED) >11.0 mmol/L (198 mg/dL), fasting plasma glucose ≥ 7.0 mmol/L (125 mg/dL) or random plasma glucose >11.0 mmol/L (Ministry of Health Malaysia, 2016). A standard lipid profile was the measurement of plasma or serum in the fasting state, and it contained four parameters. Abnormal lipid parameter measurement referred to total cholesterol >5.2 mmol/L (201 mg/dL), HDL <1.0 mmol/L (39 mg/dL) in males or 1.2 mmol/L (46 mg/dL) in females, LDL >1.8 mmol/L (70 mg/dL, established cardiovascular risk) or triglyceride >1.7 mmol/L (151 mg/dL) (Ministry of Health Malaysia, 2017).

### Statistical analyses

Data from the Malaysian National Stroke Registry were downloaded in a comma separated values format and were read and analysed using the RStudio IDE version (RStudio Team, 2015) by running the R software version 3.5.1 (survival package) (R Core Team, 2018; Therneau & Lumley, 2015). A purposeful selection of variables was done based on the crude analysis using the univariable Cox proportional hazard regression and previous literature (which was biologically or clinically important) (Bursac et al., 2008). The covariates with a significance level of less than 0.25 (two-tailed) were included manually in the process of multivariable Cox (proportional hazard) regression modelling (Hosmer, Lemeshow & Sturdivant, 2013). The results of the Wald test were examined to compare a series of the built Multivariable Cox regression models. The statistical significance level was set at 0.05 (two-tailed). The crude and adjusted hazard ratios (HR), 95% confidence intervals (CI) and corresponding *p* values were all reported.

## Results

We have analyzed the data of 8,576 first-ever stroke patients, all of whom were enrolled in the Malaysian National Stroke Registry. The primary endpoint event in this study was the first-notified recurrent stroke. A total of 394 (4.6%) subjects have had their stroke recurrence registered during the follow-up. Moreover, 95.4% of the subjects were censored at the end of the study. The mean (SD) age at first-stroke diagnosis was 61.9 (12.59). The characteristics of the study subjects are presented in Table 1, in totality and based on the recurrence status (recurred/censored).

**Table 1:** The baseline characteristics of study subjects at first-stroke event, in overall and based on the recurrence status (*n* = 8,576).

Variables	Category	Total	Overall	Recurred *n* = 394	Censored *n* = 8,182
		*n*	Mean (SD), *n*(%)	Mean (SD), *n*(%)	Mean (SD), *n*(%)
Age		8,539	61.9 (12.59)	59.9 (11.64)	62.0 (12.62)
Sex	Female	8,576	3,796 (44.3)	174 (44.1)	3,622 (44.3)
	Male		4,780 (55.7)	220 (55.8)	4,560 (55.7)
Ethnic	Malay	8,576	6,909 (80.6)	353 (89.6)	6,556 (80.1)
	Non-Malay		1,667 (19.4)	41 (10.4)	1,626 (19.9)
Stroke subtype	Intracerebral hemorrhage	8,576	1,245 (14.5)	51 (12.9)	1,194 (14.6)
	Ischemic		7,272 (84.8)	340 (86.3)	6,932 (84.7)
	Subarachnoid hemorrhage		59 (0.7)	3 (0.8)	56 (0.7)
Hypertension	No	8,576	2,658 (31.0)	93 (23.6)	2,565 (31.3)
	Yes		5,918 (69.0)	301 (76.4)	5,617 (68.7)
Diabetes Mellitus	No	8,576	5,107 (59.5)	199 (50.5)	4,908 (60.0)
	Yes		3,469 (40.5)	195 (49.5)	3,274 (40.0)
Dyslipidemia	No	8,576	6,583 (76.8)	252 (64.0)	6,331 (77.4)
	Yes		1,993 (23.2)	142 (36.0)	1,851 (22.6)
Body mass index		744	25.5 (6.75)	24.7 (4.53)	25.6 (6.79)
Glasgow coma scale (over 15)	Mild (≥13)	8,490	6,865 (80.9)	330 (84.2)	6,535 (80.7)
	Moderate (9–12)		1,238 (14.6)	51 (13.0)	1,187 (14.7)
	Severe (≤8)		387 (4.5)	11 (2.8)	376 (4.6)

**Notes.**

Abbreviations SD Standard deviation

Through the univariable Cox proportional hazard regression analysis, ten candidate variables were identified as potential prognostic factors, demonstrating both a *p*-value of less than 0.25 and clinical importance. Among these variables, we considered the fasting glucose, glucose in ED, abnormal glucose, triglyceride and total cholesterol levels as the primary variables (i.e., the variables that we hypothesized to be critical prognostic variables), whereas the included confounding variables were age, ethnicity, and the presence of hypertension, DM and dyslipidaemia. The triglyceride level, moreover, was found to be a significant prognostic variable based on the crude analysis (Table 2). Other variables (i.e., glycosylated haemoglobin, random glucose level, body mass index, abnormal HDL and LDL levels) demonstrated non-significance at *p* = 0.25.

**Table 2:** Univariable Cox (proportional hazard) regression analysis for the prognostic factors for stroke recurrence among first-ever stroke patients. Models showing regression coefficients (b), crude hazard ratios (HR) with 95% confidence intervals (CI), Wald test and the corresponding *p*-values. The outcome variable was a time-to-event variable; where the event was stroke recurrence (present or absent) and the time was the number of years to develop the event (*n* = 8,576).

Variables	Category/unit	b	Crude HR (95% CI)	Wald test	*p*-value
Age	years	−0.013	0.99 (0.98, 0.99)	11.00	<0.001^*^
HbA1c	%	0.012	1.01 (0.93, 1.10)	0.07	0.790
Glucose in ED	mmol/L	0.023	1.02 (1.00, 1.05)	3.20	0.075^*^
Random glucose	mmol/L	0.0003	1.00 (0.95, 1.05)	0	0.990
Fasting glucose	mmol/L	0.037	1.04 (1.01, 1.07)	5.80	0.016^*^
Body mass index	kg/m^2^	−0.030	0.97 (0.88, 1.08)	0.32	0.570
Sex	Female	0	1	0.10	0.750
	Male	0.032	1.03 (0.85, 1.26)		
Ethnic	Malay	0	1	16.00	<0.001^*^
	Non-Malay	0.659	1.93 (1.40, 2.67)		
Hypertension	No	0	1	9.80	0.002^*^
	Yes	0.371	1.45 (1.15, 1.83)		
Diabetes Mellitus	No	0	1	12.00	<0.001^*^
	Yes	0.350	1.42 (1.17, 1.73)		
Dyslipidemia	No	0	1	24.00	<0.001^*^
	Yes	0.510	1.66 (1.35, 2.05)		
Abnormal glucose level	No	0	1	11.00	0.001^*^
	Yes	0.377	1.46 (1.16, 1.83)		
Abnormal triglyceride	No	0	1	8.10	0.004^*^
	Yes	0.374	1.45 (1.12, 1.88)		
Abnormal TC	No	0	1	2.80	0.096^*^
	Yes	0.234	1.26 (0.96, 1.67)		
Abnormal HDL	No	0	1	0.10	0.750
	Yes	−0.043	0.96 (0.73, 1.25)		
Abnormal LDL	No	0	1	0.37	0.540
	Yes	0.174	1.19 (0.68, 2.08)		

**Notes.**

HR, Hazard ratio = exp (*β*); 95% CI exp(*β*-1.965*SE(*β*)), exp(*β*+1.965*SE(*β*)).

* level of significance, *p* = 0.25.

Abbreviations HbA1c Glycosylated hemoglobin ED Emergency department TC Total cholesterol HDL High-density lipoprotein (cholesterol) LDL Low-density lipoprotein (cholesterol)

Four models were built using the multivariable Cox (proportional hazard) regression analysis to explain if the glucose profile was an independent prognostic factor for stroke recurrence when other confounders were adjusted. Since the primary variable, glucose profile, is often related to the premorbid diagnosis of DM, the DM diagnosis was the first covariate to be controlled. Table 3 presents a series of multivariable Cox regression models that included the glucose parameters and DM diagnosis. The glucose profile was consistently not associated with an increased risk of stroke recurrence when DM was adjusted.

**Table 3:** Multivariable Cox (proportional hazard) regression analysis for glucose profile prognosticating stroke recurrence among first-ever stroke patients in Malaysia. Models show estimated regression coefficient (b), standard error (SE), adjusted hazard ratios (Adj HR) with 95% confidence intervals (CI) and the corresponding *p*-values (*n* = 8,576). The outcome variable was a time-to-event variable; where the event was stroke recurrence (present or absent) and the time was the number of years to develop the event. Model 1 is the crude model with only one primary variable (abnormal glucose level).

Variables	Model 1^a^ (*n* = 7,123)		Model 2 (*n* = 7,123)		Model 3 (*n* = 4,729)		Model 4 (*n* = 3,355)	
	b (SE)	Crude HR (95% CI)	b (SE)	Adj HR (95% CI)	b (SE)	Adj HR (95% CI)	b (SE)	Adj HR (95% CI)
No. of events	310		310		170		179	
Abnormal glucose level	0.38 (0.12)	1.46^*^ (1.16, 1.83)	0.25 (0.13)	1.28 (1.00, 1.65)				
Fasting glucose							0.02 (0.02)	1.02 (0.99, 1.06)
Glucose in ED					0.01 (0.02)	1.01 (0.98, 1.04)		
Diabetes mellitus			0.28 (0.13)	1.33^*^ (1.04, 1.70)	0.38 (0.17)	1.46^*^ (1.05, 2.04)	0.37 (0.16)	1.44^*^ (1.05, 1.98)

**Notes.**

HR, Hazard Ratio = exp (*β*); 95% CI = exp(*β*-1.965*SE(*β*)), exp(*β*+1.965*SE(*β*)).

a Unadjusted model

* level of significance, *p* = 0.05.

Abbreviations ED Emergency Department

Interactions were unlikely for the adjusted models. Scaled and non-scaled Schoenfeld residuals test were applied to check the proportional hazard assumption of the model.

In a series of the multivariable Cox regression models that included the total cholesterol variable and one additional variable (i.e., dyslipidemia, hypertension, DM and ethnic), the total abnormal cholesterol level was not associated with an increased risk of stroke recurrence (Table 4). The triglyceride level, moreover, was found to be a significant prognostic variable based on the crude analysis. Triglyceride level could significantly prognosticate stroke recurrence at the crude HR stood at 1.45 (95% CI [1.22–1.88]) and the adjusted HR at 1.36 (95% CI [1.05–1.77]) together with dyslipidemia was adjusted in TG model 1; adjusted HR at 1.34 (95% CI [1.03–1.73]) when dyslipidemia and hypertension were adjusted in TG model 2 (Table 5). After adjustment for DM (TG model 3 in Table 5), an abnormal triglyceride level did not longer reach statistical significance in prognosticating stroke recurrence.

**Table 4:** Multivariable Cox (proportional hazard) regression analysis for total cholesterol prognosticating stroke recurrence among first-ever stroke patients in Malaysia. Models show estimated regression coefficient (b), standard error (SE), adjusted hazard ratios (Adj HR) with 95% confidence intervals (CI) and the corresponding *p*-values (*n* = 8,576). The outcome variable was a time-to-event variable; where the event was stroke recurrence (present or absent) and the time was the number of years to develop the event.

Variables	TC Model 1 (*n* = 5,062)		TC Model 2 (*n* = 5,062)		TC Model 3 (*n* = 5,062)		TC Model 4 (*n* = 5,043)	
	b (SE)	Adj HR (95% CI)	b (SE)	Adj HR (95% CI)	b (SE)	Adj HR (95% CI)	b (SE)	Adj HR (95% CI)
Events	241		241		241		240	
Abnormal TC	0.04 (0.15)	1.04 (0.77, 1.38)	0.23 (0.14)	1.25 (0.95, 1.65)	0.23 (0.14)	1.26 (0.96, 1.66)	0.19 (0.14)	1.19 (0.90, 1.57)
Dyslipidemia	0.65 (0.14)	1.92* (1.47, 2.50)						
Hypertension			0.44 (0.15)	1.55* (1.15, 2.09)				
Diabetes Mellitus					0.37 (0.13)	1.45* (1.13, 1.87)		
Ethnic							0.60 (0.22)	1.81* (1.18, 2.80)

**Notes.**

HR, Hazard Ratio = exp (*β*); 95% CI = exp(*β*-1.965*SE(*β*)), exp(*β*+1.965*SE(*β*)).

* level of significance, *p* = 0.05.

Abbreviations TC Total cholesterol

Interactions were unlikely.

Scaled and non-scaled Schoenfeld residuals test were applied to check the proportional hazard assumption of the model.

**Table 5:** Multivariable Cox (proportional hazard) regression analysis for triglyceride prognosticating stroke recurrence among first-ever stroke patients in Malaysia. Models show estimated regression coefficient (b), standard error (SE), adjusted hazard ratios (Adj HR) with 95% confidence intervals (CI) and the corresponding *p*-values (*n* = 8,576). The outcome variable was a time-to-event variable; where the event was stroke recurrence (present or absent) and the time was the number of years to develop the event.

Variables	TG Model 1 (*n* = 5,074)		TG Model 2 (*n* = 5,074)		TG Model 3 (*n* = 5,074)	
	b (SE)	Adj HR (95% CI)	b (SE)	Adj HR (95% CI)	b (SE)	Adj HR (95% CI)
Events	246		246		246	
Abnormal triglyceride	0.31 (0.13)	1.36* (1.05, 1.77)	0.29 (0.13)	1.34^*^ (1.03, 1.73)	0.25 (0.14)	1.28 (0.98, 1.66)
Dyslipidemia	0.61 (0.13)	1.84^*^ (1.43, 2.37)	0.58 (0.13)	1.78^*^ (1.39, 2.30)	0.58 (0.13)	1.78^*^ (1.38, 2.30)
Hypertension			0.36 (0.15)	1.43^*^ (1.06, 1.92)		
Diabetes Mellitus					0.31 (0.13)	1.36^*^ (1.05, 1.76)

**Notes.**

HR, Hazard Ratio = exp (*β*); 95% CI = exp(*β*-1.965*SE(*β*)), exp(*β*+1.965*SE(*β*)).

* level of significance, *p* = 0.05

Interactions were unlikely.

Scaled and non-scaled Schoenfeld residuals test were applied to check the proportional hazard assumption of the model.

## Discussion

As noted previously, the impact of diabetes mellitus (Chandratheva, Geraghty & Rothwell, 2011; Ghandehari et al., 2013; Li et al., 2017; Long et al., 2016) and dyslipidemia (Callaly et al., 2016; Feng et al., 2009; Ghandehari et al., 2013; Kumral et al., 2014) on the prognosis of stroke recurrence has been studied before, but the evidence on this relationship remains inconclusive. To our knowledge, the present research is the first relatively large, multicenter, retrospective cohort study to confirm the prognostication of glucose and lipid profiles on stroke recurrence among first-ever stroke patients in Malaysia. These findings are useful for secondary prevention efforts. The present study demonstrated three key findings: first, the glucose profile or abnormal glucose level could not prognosticate stroke recurrence; second, the total cholesterol level could not prognosticate stroke recurrence; and third, the triglyceride level could independently prognosticate stroke recurrence.

Contrary to previous findings, we found no association between impaired glucose metabolism and stroke recurrence in the time-to-event analysis. When comparing our results to previous studies, it is notable that such paradoxical results might have occurred due to the use of different operational definitions. For example, in the present and previous studies, the timing of the fasting blood samples obtained during/after the acute stroke event was varied, the model specifications were dissimilar, and the conducted statistical analyses diverged (i.e., Cox versus logistic regression) (Ghandehari et al., 2013; Liu et al., 2015; Mi et al., 2012). The present study showed that the comorbidity of diabetes mellitus affected the prognostication of impaired glucose metabolism for stroke recurrence. It is difficult to explain such results if diabetes mellitus is a mediator, and it is challenging to establish whether the disturbances of glucose metabolism constitute a stress response to the acute neurological insult or an indication of underlying diabetes (Matz et al., 2006; Vancheri et al., 2005).

In the present study, comorbid dyslipidemia was a reliable prognosticator of stroke recurrence. A similar conclusion was reached in studies conducted abroad, including the West China Hospital stroke registry-based study (Feng et al., 2009), the Ege stroke registry-based study in Turkey (Kumral et al., 2014), and a population stroke study in North Dublin (Callaly et al., 2016). Hence, this justifies the inclusion of dyslipidemia as a confounding factor in the multivariable analysis. The weak prognostication of HDL and stroke recurrence also confirms the results of a previous study (Liu et al., 2015). The recurrence risk associated with higher plasma total cholesterol levels disappeared after adjusting for comorbid dyslipidemia. At this stage of understanding, we believe that total cholesterol and dyslipidemia confound each other. This is because, from our observations and experience in Malaysia, physicians tend to diagnose dyslipidemia based on the total cholesterol level but not other lipid parameters, and a similar phenomenon has been noted in China (Feng et al., 2009).

Concerning the implicative lipid profile parameters for stroke recurrence, we have demonstrated that the triglyceride level consistently confers an increased risk of stroke recurrence, when adjusting for dyslipidemia and hypertension. Recent randomized, double-blinded clinical trials showed that fasting plasma triglyceride level predicted both short-term and long-term cardiovascular risk, despite the background statin treatment occurring in patients with the acute coronary syndrome. Moreover, this association remained significant after adjusting for conventional risk factors usually associated with the triglyceride level (Schwartz et al., 2015). However, our finding is in contrast with that from a single hospital-based study with a three-month follow-up period (Liu et al., 2015)—a difference which can perhaps be attributed to the cohort duration. On top of that, our data addressed the weak prognostication of triglyceride level to stroke recurrence after adjusting for the comorbidity diabetes mellitus. Under certain assumptions, this can be construed as the unaccountable effect of insulin resistance in the relationship between triglyceride level and cardiovascular risk (Schwartz et al., 2015; Taskinen, 2005; Van de Woestijne et al., 2013), in which insulin resistance played an important role in the pathogenic basis of atherogenic dyslipidemia (Zhao et al., 2015). In short, we believe that our finding indicates a need to encourage physicians to emphasize triglyceride monitoring, and we recommend marking the triglyceride level as a potential treatment target, particularly in nondiabetic stroke patients.

Our results also offer several clinical implications for current stroke management. Impaired glucose metabolism has no prognostic effect on stroke recurrence. However, stroke patients with abnormal glucose level on admission and previously undiagnosed diabetes should be tested with a simple, inexpensive oral glucose tolerance test at discharge (i.e., in the post-acute phase). Furthermore, an oral glucose tolerance test is a better predictor of cardiovascular events than a fasting plasma glucose evaluation, and those with abnormal glucose metabolism without a previous diagnosis of diabetes mellitus are more insulin-resistant than those with a healthy glucose metabolism (Vancheri et al., 2005). This suggestion was made in the past based on the finding that diabetic patients experience a relatively more severe stroke and a poorer prognosis when compared to nondiabetic patients (Matz et al., 2006).

One significant repercussion of the present study relates to the emphasis on the prognostication of triglyceride level on stroke recurrence, whereby insufficient attention has been paid in our clinical practices when patients were admitted for their first stroke event. Per previous suggestions (Schwartz et al., 2015; Talayero & Sacks, 2011), nonpharmacological management and medication prescriptions should be initiated in Malaysia stroke populations, as per the clinical practice guidelines (Ministry of Health Malaysia, 2017). Maybe nutritional counselling could also be offered to stroke patients during hospitalization so they could make alterations in their diet that could influence (i.e., reverse or attenuate) their atherogenic dyslipidemia (Musunuru, 2010). It is commonly argued that the cholesterol content of triglyceride-rich remnant lipoproteins confers atherogenicity, thus reflecting the burden of hypertriglyceridemia on the vascular risk, despite the background risk factors being effectively treated and other lipid levels being at an ideal level (Feingold & Grunfeld, 2018; International Chair on Cardiometabolic Risk, 2019; Schwartz et al., 2015; Van de Woestijne et al., 2013). Furthermore, the inflammatory component associated with remnant cholesterol contributed to the atherosclerosis progression (Varbo & Nordestgaard, 2016).

## Limitations

The present study is constrained by the shortcomings of the registry (i.e., the source of secondary data), the quality and accuracy of the data, as well as a possibly occurring information bias (Gliklich, Dreyer & Leavy, 2014; Krumholz, 2009). An incomplete follow-up after the first-stroke event might have resulted in misclassification and a potentially underestimated recurrence event. Nevertheless, this pitfall is unlikely to cause a significant problem, as survival analysis can account for censoring, wherein other outcomes (e.g., death, loss to follow-up) were treated as censored observations (George, Seals & Aban, 2014). The second limitation relates to the variable of selection in building prognostic models, such as the presence of cardiac disease (including atrial fibrillation and the use of anti-coagulant therapy), quality of life, and the use of complementary and alternative medicines post-stroke that were not recorded in the registry (Kadir, Hamid & Mohammad, 2015; Rachpukdee et al., 2013). We also did not model the serial measurement of glucose and lipid profiles from post-discharge to the recurrent stroke event, even though the incorporation of serial covariate measurements was recommended in time-to-event analysis through the analysis of the joint model (Ibrahim, Chu & Chen, 2010). The extent of the degree to which the exclusion of these variables could influence the prognostication is indefinite, and we included the majority of the important covariates that were recognized to our literature search.

## Conclusion

Out of all parameters in the glucose and lipid profiles, the triglyceride level was revealed as a consistent prognostic factor for stroke recurrence in first-ever stroke patients. This finding supports the hypothesis that having an abnormal triglyceride level at the time of the first stroke event signals a predisposition for stroke recurrence and emphasizes the contribution that triglyceride treatment and monitoring may play in tertiary stroke prevention. Consistent with the recommendations mentioned above, we suggest that further investigations should be conducted in first-ever stroke patients with impaired glucose and lipid profiles, and we call for further discussion on lifestyle changes and the development of therapeutic interventions that will target all lipid markers including hypertriglyceridemia.

## Additional Information and Declarations

### Data Availability

The following information was supplied regarding data availability: This medical research uses identifiable human material and individual data. The institutional review board from both the Human Research and Ethics Committee (HREC) of Universiti Sains Malaysia (USM/JEPeM/1711-576) and Malaysia Research and Ethics Committee (MREC), Ministry of Health Malaysia (NMRR-17-2500-38534) has specific legal requirements to prohibit public sharing of the dataset. HREC: jepem@usm.my (Mr. Bazlan), Human Research Ethics Committee USM, Division of Research and Innovation, USM health campus, 16150 Kubang Kerian, Kelantan. MREC: leekengyee@crc.moh.gov.my (Dr Lee Keng Yee). Medical Research and Ethics Committee Secretariat, National Institutes of Health, Persiaran Setia Murni, Setia Alam, 40170 Shah Alam.

## References

[ref-1] Awada A (2011). Primary and secondary prevention of ischemic stroke. Journal Medical Libanais. Lebanese Medical Journal.

[ref-2] Bursac Z, Gauss CH, Williams DK, Hosmer DW (2008). Purposeful selection of variables in logistic regression. Source Code for Biology and Medicine.

[ref-3] Callaly E, Ni Chroinin D, Hannon N, Marnane M, Akijian L, Sheehan O, Merwick A, Hayden D, Horgan G, Duggan J, Murphy S, O’Rourke K, Dolan E, Williams D, Kyne L, Kelly PJ (2016). Rates, predictors, and outcomes of early and late recurrence after stroke: the North Dublin population stroke study. Stroke.

[ref-4] Chandratheva A, Geraghty OC, Rothwell PM (2011). Poor performance of current prognostic scores for early risk of recurrence after minor stroke. Stroke.

[ref-5] Counsell C, Dennis M (2001). Systematic review of prognostic models in patients with acute stroke. Cerebrovascular Diseases.

[ref-6] Feingold KR, Grunfeld C (2018). Introduction to lipids and lipoproteins. Endotext [Internet].

[ref-7] Feng SJ, Liu M, Li WZ, Li W, Zhang SH (2009). A prospective study of stroke recurrence and the risk factors. Nan Fang Yi Ke Da Xue Xue Bao.

[ref-8] GBD 2015 Risk Factors Collaborators (2016). Global, regional, and national comparative risk assessment of 79 behavioural, environmental and occupational, and metabolic risks or clusters of risks, 1990–2015: a systematic analysis for the Global Burden of Disease Study 2015. The Lancet.

[ref-9] George B, Seals S, Aban I (2014). Survival analysis and regression models. Journal of Nuclear Cardiology.

[ref-10] Ghandehari K, Khajedaluei MR, Yazdankhah Z, Ghandehari K (2013). Risk factors of short-term stroke recurrence in patients with minor ischemic cerebrovascular events. ARYA Atherosclerosis.

[ref-11] Gliklich RE, Dreyer NA, Leavy MB (2014). Registries for evaluating patient outcomes: a user’s guide [Internet].

[ref-12] Hosmer DW, Lemeshow S, Sturdivant RX (2013). Applied logistic regression.

[ref-13] Ibrahim JG, Chu H, Chen LM (2010). Basic concepts and methods for joint models of longitudinal and survival data. Journal of Clinical Oncology.

[ref-14] International Chair on Cardiometabolic Risk (2019). Atherogenic Dyslipidemia. http://www.myhealthywaist.org/the-concept-of-cmr/intra-abdominal-adipose-tissue-the-culprit/complications-of-intra-abdominal-obesity/atherogenic-dyslipidemia/print.html?printebook=true&cHash=5205fa63b2.

[ref-15] Jia Q, Zhao X, Wang C, Wang Y, Yan Y, Li H, Zhong L, Liu L, Zheng H, Zhou Y (2011). Diabetes and poor outcomes within 6 months after acute ischemic stroke. Stroke.

[ref-16] Kadir AA, Hamid AH, Mohammad M (2015). Pattern of complementary and alternative medicine use among Malaysian stroke survivors: a hospital-based prospective study. Journal of Traditional and Complementary Medicine.

[ref-17] Krumholz HM (2009). Registries and selection bias: the need for accountability. Circulation: Cardiovascular Quality and Outcomes.

[ref-18] Kumral E, Evyapan D, Gokcay F, Karaman B, Orman M (2014). Association of baseline dyslipidemia with stroke recurrence within five-years after ischemic stroke. International Journal of Stroke.

[ref-19] Kutner MH, Nachtsheim CJ, Neter J, Li W (2004). Applied linear statistical models.

[ref-20] Li F, Yang L, Yang R, Xu W, Chen FP, Li N, Zhang JB (2017). Ischemic stroke in young adults of Northern China: characteristics and risk factors for recurrence. European Neurology.

[ref-21] Liu L, Zhan L, Wang Y, Bai C, Guo J, Lin Q, Liang D, Xu E (2015). Metabolic syndrome and the short-term prognosis of acute ischemic stroke: a hospital-based retrospective study. Lipids in Health and Disease.

[ref-22] Long X, Lou Y, Gu H, Guo X, Wang T, Zhu Y, Zhao W, Ning X, Li B, Wang J, An Z (2016). Mortality, recurrence, and dependency rates are higher after acute ischemic stroke in elderly patients with diabetes compared to younger patients. Frontiers in Aging Neuroscience.

[ref-23] Matz K, Keresztes K, Tatschl C, Nowotny M, Dachenhausen A, Brainin M, Tuomilehto J (2006). Disorders of glucose metabolism in acute stroke patients. An underrecognized problem. Diabetes Care.

[ref-24] Mi D, Jia Q, Zheng H, Hoff K, Zhao X, Wang C, Liu G, Wang Y, Liu L, Wang X, Wang Y (2012). Metabolic syndrome and stroke recurrence in Chinese ischemic stroke patients—the ACROSS-China study. PLOS ONE.

[ref-25] Ministry of Health Malaysia (2016). Clinical practice guidelines: management of type 2 diabetes mellitus 5th edition.

[ref-26] Ministry of Health Malaysia (2017). 5th edition of clinical practice guidelines: management of Dyslipidaemia 2017.

[ref-27] Musunuru K (2010). Atherogenic dyslipidemia: cardiovascular risk and dietary intervention. Lipids.

[ref-28] Nazifah SN, Azmi IK, Hamidon BB, Looi I, Zariah AA, Hanip MR (2012). National stroke registry (NSR): Terengganu and Seberang Jaya experience. Medical Journal of Malaysia.

[ref-29] NNEUR (2013). Overview of the national neurology registry. http://acrm.org.my/nneur/overview.php.

[ref-30] Park JH, Lee J, Ovbiagele B (2014). Nontraditional serum lipid variables and recurrent stroke risk. Stroke.

[ref-31] R Core Team (2018). https://www.R-project.org/.

[ref-32] Rachpukdee S, Howteerakul N, Suwannapong N, Tang-Aroonsin S (2013). Quality of life of stroke survivors: a 3-month follow-up study. Journal of Stroke and Cerebrovascular Diseases.

[ref-33] RStudio Team (2015). http://www.rstudio.com/.

[ref-34] Schwartz GG, Abt M, Bao W, DeMicco D, Kallend D, Miller M, Mundl H, Olsson AG (2015). Fasting triglycerides predict recurrent ischemic events in patients with acute coronary syndrome treated with statins. Journal of the American College of Cardiology.

[ref-35] Singer JD, Willett JB (2003). Applied longitudinal data analysis: modeling change and event occurrence.

[ref-36] Talayero BG, Sacks FM (2011). The role of triglycerides in atherosclerosis. Current Cardiology Reports.

[ref-37] Taskinen M-R (2005). Type 2 diabetes as a lipid disorder. Current Molecular Medicine.

[ref-38] Therneau T, Lumley T (2015). A package for survival analysis in S.

[ref-39] Vancheri F, Curcio M, Burgio A, Salvaggio S, Gruttadauria G, Lunetta MC, Dovico R, Alletto M (2005). Impaired glucose metabolism in patients with acute stroke and no previous diagnosis of diabetes mellitus. QJM.

[ref-40] Varbo A, Nordestgaard BG (2016). Remnant cholesterol and triglyceride-rich lipoproteins in atherosclerosis progression and cardiovascular disease. Arteriosclerosis, Thrombosis, and Vascular Biology.

[ref-41] Van de Woestijne AP, Wassink AM, Monajemi H, Liem AH, Nathoe HM, Van der Graaf Y, Visseren FL (2013). Plasma triglyceride levels increase the risk for recurrent vascular events independent of LDL-cholesterol or nonHDL-cholesterol. International Journal of Cardiology.

[ref-42] World Health Organization (2015). International statistical classifcation of diseases and related health problems—10th revision.

[ref-43] Zhao L, Wang R, Song B, Tan S, Gao Y, Fang H, Lu J, Xu Y (2015). Association between atherogenic dyslipidemia and recurrent stroke risk in patients with different subtypes of ischemic stroke. International Journal of Stroke.

